# Systemic and coronary levels of CRP, MPO, sCD40L and PlGF in patients with coronary artery disease

**DOI:** 10.1186/s13104-015-1677-8

**Published:** 2015-11-14

**Authors:** Siew Wai Fong, Ling Ling Few, Wei Cun See Too, Boon Yin Khoo, Nik Nor Izah Nik Ibrahim, Shaiful Azmi Yahaya, Zurkurnai Yusof, Rosli Mohd Ali, Abdul Rashid Abdul Rahman, Get Bee Yvonne-Tee

**Affiliations:** School of Health Sciences, Universiti Sains Malaysia, 16150 Kubang Kerian, Kelantan Malaysia; Institute for Research in Molecular Medicine (INFORMM), Universiti Sains Malaysia, 11800 Penang, Malaysia; School of Medical Sciences, Universiti Sains Malaysia, 16150 Kubang Kerian, Kelantan Malaysia; National Heart Institute, 50400 Kuala Lumpur, Malaysia; Cyberjaya University College of Medical Sciences, 63000 Cyberjaya, Selangor Malaysia

**Keywords:** Biomarker, Coronary artery disease, Coronary circulation, Systemic circulation

## Abstract

**Background:**

Biomarkers play a pivotal role in the diagnosis and management of patients with acute coronary syndrome. This study aimed to investigate the differences in level of several biomarkers, i.e. C-reactive protein, myeloperoxidase, soluble CD40 ligand and placental growth factor, between acute coronary syndrome and chronic stable angina patients. The relationship between these biomarkers in the coronary circulation and systemic circulation was also investigated.

**Methods:**

A total of 79 patients were recruited in this study. The coronary blood was sampled from occluded coronary artery, while the peripheral venous blood was withdrawn from antecubital fossa. The serum concentrations of C-reactive protein, soluble CD40 ligand and placental growth factor and plasma concentration of myeloperoxidase were measured using ELISA method.

**Results:**

The systemic level of the markers measured in the peripheral venous blood was significantly increased in acute coronary syndrome compared to chronic stable angina patients. The concentrations of the C-reactive protein, myeloperoxidase and soluble CD40 ligand taken from peripheral vein were closely similar to the concentration found in coronary blood of ACS patients. The level of placental growth factor was significantly higher in coronary circulation than its systemic level.

**Conclusion:**

The concentration of these C-reactive protein, myeloperoxidase, soluble CD40 ligand and placental growth factor were significantly increased in acute coronary syndrome patients. The concentration of the markers measured in the systemic circulation directly reflected those in the local coronary circulation. Thus, these markers have potential to become a useful tool in predicting plaque vulnerability in the future.

## Background

Acute coronary syndrome (ACS) refers to a spectrum of clinical manifestations, which includes disruption of coronary plaque, thrombosis, embolization and varying degrees of perfusion obstruction. The understanding of the events in pathophysiology of ACS contributes to the emergence of several biomarkers such as myeloperoxidase (MPO), soluble CD40 ligand (sCD40L) and placental growth factor (PlGF).

Myeloperoxidase is released from neutrophilic granules and monocytes during inflammation [1]. It plays a pivotal role in plaque instability by causing fibrous cap disintegration. It catalyzes the formation of hypochlorite from chloride and hydrogen peroxide. Its end product hypochlorous acid, HOCl, activates matrix metalloproteinases (MMPs) [2] and deactivates inhibitors of MMPs [3]. Thus, it promotes weakening of the fibrous cap and results in the formation of vulnerable atherosclerotic plaque. Besides MPO, the platelet-derived sCD40L also plays an essential role in plaque destabilization. It stimulates vascular endothelial cells inflammation by the secretion of chemokines and cytokines [4]. Membrane bound CD40L and sCD40L interact with the CD40 receptor molecule on B cells, monocytes, macrophages, endothelial and smooth muscle cells in atheroma. This leads to the release of MMPs which subsequently destabilize the plaque [5]. The PlGF is a platelet-derived protein that has a high homology and functions similar to vascular endothelial growth factor. It involves in the regulation of vascular endothelial growth by recruiting monocytes [6]. Moreover, PlGF might form a heterodimer with vascular endothelial growth factor, thereby enhancing pro-atherogenic effects of this growth factor by either reducing the bioavailability of vascular endothelial growth factor (VEGF) or, at the other extreme, by increasing the activity of low concentrations of VEGF [7].

There were studies reporting on the elevated expression of MPO, sCD40L and PlGF in ACS patients [8–10]. Most of these studies measured the concentration of markers in the systemic circulation. The researchers generally presumed that the elevation of markers sampled from the peripheral circulation is a reflection of the release of these markers from the ruptured coronary plaque. However, there is a possibility that the markers are released from non-coronary sources, which subsequently transform the stable plaque into unstable form. Therefore, this study aimed to measure the systemic and coronary levels of MPO, sCD40L, PlGF together with C-reactive protein (CRP), in patients with ACS who underwent a coronary angiogram and/or percutaneous intervention as clinically indicated compared to chronic stable angina (CSA) patients scheduled for elective angioplasty.

## Methods

### Participants

The ethical approval was obtained from Human Research Ethics Committee of Universiti Sains Malaysia and National Heart Institute, Malaysia prior to study implementation. All participants agreed to participate and signed the written informed consent. A total of 39 patients with ACS and 40 patients with CSA were recruited. The ACS cases included ST elevation myocardial infarction (STEMI), non ST elevation myocardial infarction (NSTEMI) and unstable angina (UA). Patients are defined as STEMI when they present with biomarkers of myocardial necrosis (i.e. typical rise and gradual fall of troponin or more rapid rise and fall of creatine kinase MB) and at least one of the following criteria: clinical history of ischemic type chest pain; development of pathologic Q waves on the electrocardiogram (ECG); ECG changes indicative of ischemia. Patients are defined as UA/NSTEMI when they present with three possible types of chest pain [i.e. new onset (2 months) angina that is severe and/or frequent (>3 episodes per day), accelerating angina and/or angina at rest] with ECG changes (ST segment depression >0.05 mV or T-wave inversion >0.2 mV in the precordial leads). NSTEMI is differentiated from UA by the presence of elevated serum cardiac markers. All ACS patients underwent a coronary angiogram within 7 days after admission, and an angiogram was routinely scheduled for CSA patients.

### Blood sampling and processing

The blood sample was collected from ACS patient within 48 h after onset of symptom. The coronary blood sample was drawn from the culprit artery for ACS patients and the most severely stenotic artery for CSA patients. On the identification of the lesion, 100 IU/kg heparin was administered intravenously during the procedure. In ACS patients, the culprit coronary artery was engaged with guiding catheter and subsequently wired with guide wire. An export aspiration catheter was advanced a few centimeters distal to the culprit lesion after crossing the culprit lesion. Aspiration of 10 mL of intracoronary blood was done with a syringe from the distal end of the lesion ensued. Patients were excluded from the study if the aspiration catheter failed to pass the culprit lesion for any reason. In CSA patients, intracoronary blood was drawn from the coronary artery at the time of guiding catheter engagement into the coronary ostium of the most severe stenotic artery. Balloon angioplasty and stent implantation were then performed according to standard techniques. Another 10 mL of whole blood sample was drawn from a peripheral vein (antecubital fossa) after the angioplasty procedure. The blood samples were collected in both plain tube and EDTA tube in order to obtain serum and plasma samples after centrifugation. The samples were then stored at −80 °C until further analysis.

### Measurement of MPO, sCD40L, PlGF and CRP

Serum sCD40L and PlGF levels were measured using commercially available enzyme-linked immunosorbent assay kits (R&D Systems, Abingdon, UK). Serum CRP level was assessed using a commercial ELISA kit from DRG diagnostics (DRG International, USA). Quantitative measurement of MPO in plasma was performed using optimized in-house ELISA method adopted from a previous study [11]. In general, ELISA was run with plates pre-coated with antibodies. Standards, samples, and a conjugated detection antibody were added to each well. A horseradish peroxidase/3,3′,5,5′-tetramethylbenzidine-system was used for the enzymatic color reaction. Plates were read at 450 nm on ELISA reader (Thermo Scientific, USA). Protein concentrations were calculated by comparing optical density (OD) values of samples with OD of known concentrations of the standards prepared by the kit’s manufacturer. The intra-assay CVs (CRP 3.0 %, MPO 1.6 %, sCD40L 2.9 %, PlGF 2.4 %) and inter-assay CVs (CRP 6.1 %, MPO 5.0 %, sCD40L 6.2 %, PlGF 11.2 %) were calculated.

### Statistical analysis

Statistical analysis was performed using SPSS software version 18 (SPSS Inc., Chicago, IL, USA). Data were presented as mean ± SD However, if the data were not normally distributed the median and inter-quartile range were used. Data normality was verified using the Shapiro–Wilks test. Independent T-tests (parametric) or Mann–Whitney U test (non-parametric) was used to study the difference between groups (ACS vs. CSA) and sampling sites (coronary vs. systemic). Categorical data were compared using the Chi square test and fisher’s exact test. A *p* value < 0.05 was considered statistically significant. The association between the marker’s level and the incidence of ACS was investigated using multivariate logistic regression to calculate the adjusted odds ratio with 95 % confidence interval (CI).

## Results

### Baseline characteristics of participants

Table 1 presents baseline clinical characteristics of patients in this study. Twenty-two ACS patients suffered from multiple-vessel disease instead of a single vessel disease. The prevalence of diabetes mellitus, hyperlipidaemia, smoking, and statin use were similar in both study groups. There was no significant difference in age and gender between groups. However, there were significantly more CSA patients suffered from myocardial infarction previously compared to ACS patients.

**Table 1:** Baseline clinical characteristics of the study groups

Variables	Acute coronary syndrome (*n* = 39)	Chronic stable angina (*n* = 40)	*p* value
Sex ratio (male:female)	37:1	37:1	1.000
Age, years median (IQR)	53 (43–64)	46 (42–61)	0.806
Multiple vessel disease (*n*, %)	22 (56.4 %)	30 (75.0 %)	0.082
Hypertension (*n*, %)	23 (59.0 %)	32 (80.0 %)	0.042*
Diabetes mellitus (*n*, %)	17 (43.6 %)	22 (55.0 %)	0.311
Hyperlipidaemia (*n*, %)	26 (66.7 %)	27 (67.5 %)	0.937
Cholesterol (mmol/L) median (IQR)	4.8 (1.0)	4.7 (1.1)	0.562
LDL cholesterol (mmol/L)	2.6 (2.1–3.6)	2.7 (1.9–3.4)	0.456
Triglycerides (mmol/L) median (IQR)	1.4 (1.1–2.3)	1.8 (1.7–2.5)	0.006*
Platelet count (×10^9^/L) (mean ± SD)	231.0 ± 60.1	250.0 ± 58.6	0.177
Creatinine (µmol/L) median (IQR)	107 (99–119)	99 (84–106)	0.023*
Statin (*n*, %)	26 (66.7 %)	27 (67.5 %)	0.937
Smoking (*n*, %)	14 (35.9 %)	16 (40.0 %)	0.707
Previous myocardial infarction (*n*, %)	2 (5.1 %)	14 (35.0 %)	0.001*

Statistic data were given as median (IQR), mean ± SD or *n*, %

*IQR* interquartile range, *SD* standard deviation

* Significant with *p* < 0.05

### Systemic levels of CRP, MPO, PlGF and sCD40L

Systemic venous levels of CRP, MPO, PlGF and sCD40L in both CSA and ACS groups are shown in Table 2. Concentrations of the four markers showed significant differences between CSA and ACS patients. The CRP, MPO, PlGF and sCD40L were found associated with ACS independently, after adjusted for variables which are known to have an effect on marker levels (i.e. age, gender, smoking and statin use) (Table 3).

**Table 2:** Systemic levels of CRP, MPO, sCD40L and PlGF in both CSA and ACS patients

	CRP (mg/L)	MPO (ng/mL)	sCD40L (ng/mL)	PlGF (pg/mL)
ACS	22.03 (4.72–45.86)	529.53 (323.91–1015.33)	2.95 (1.31–7.14)	42.36 (23.72–59.64)
CSA	3.97 (2.39–8.38)	426.06 (351.28–511.93)	1.84 (0.90–3.15)	16.28 (6.63–36.44)
*p* value	<0.001*	0.023*	0.038*	<0.001*

Data were given as median (IQR)

*CRP* C-reactive protein, *MPO* myeloperoxidase, *sCD40L* soluble CD40 ligand, *PlGF* placental growth factor

* Significant with *p* < 0.05, tested by Mann–Whitney U test

**Table 3:** List of markers and their associations with the incidence of ACS analyzed using multivariate logistic regression

Biomarkers	Regression coefficient	Adjusted odds ratio (95 % CI)^a^	*p* value
CRP (mg/L)	0.102	1.107 (1.038–1.181)	0.002*
sCD40L (ng/mL)	0.195	1.216 (1.037–1.425)	0.016*
PlGF (pg/mL)	0.023	1.023 (1.006–1.041)	0.009*
MPO (ng/mL)	0.001	1.001 (1.000–1.003)	0.039*

* Significant at *p* value < 0.05

^a^Adjusted with age, gender, smoking and statin use

### Comparison between systemic and coronary markers levels

In patients with ACS, there was no significant difference between systemic and coronary blood levels of CRP, MPO and sCD40L. However, the level of PlGF in coronary circulation was significantly higher than its level in systemic circulation (Table 4). The coronary levels of all markers established significant positive correlations with their levels in systemic circulation (Table 4).

**Table 4:** Systemic and coronary levels of CRP, MPO, sCD40L and PlGF in both CSA and ACS patients

	CRP (mg/L)	MPO (ng/mL)	sCD40L (ng/mL)	PlGF (pg/mL)
CSA				
Coronary	3.14 (1.60–6.40)	442.88 (256.38–525.20)	1.90 (1.34–3.77)	51.57 (8.85–81.66)
Systemic	3.97 (2.39–8.38)	426.06 (351.28–511.93)	1.84 (0.90–3.15)	16.28 (6.63–36.44)
*p* value^a^	0.001*	0.192	0.038*	<0.001*
Spearman’s ρ	0.904	0.427	0.433	0.663
*p* value^b^	<0.001*	0.006*	0.005*	<0.001*
ACS				
Coronary	16.34 (4.93–40.12)	608.75 (283.54–998.22)	3.19 (1.66–6.60)	53.43 (37.72–74.69)
Systemic	22.03 (4.72–45.86)	529.53 (323.91–1015.33)	2.95 (1.31–7.14)	42.36 (23.72–59.54)
*p* value^a^	0.199	0.567	0.759	0.009*
Spearman’s ρ	0.961	0.818	0.597	0.475
*p* value^b^	<0.001*	<0.001*	<0.001*	0.002*

*CRP* C-reactive protein, *MPO* myeloperoxidase, *sCD40L* soluble CD40 ligand, *PlGF* placental growth factor

Data were given as median (IQR)

* Significant with *p* < 0.05

^a^ *p*: *p* value for the Wilcoxon signed-ranks test

^b^ *p*: *p* value of the correlation test

### Correlation between coronary markers levels

We did correlation test to study the coronary relationship between CRP, MPO, sCD40L and PlGF in ACS patients. The correlation between coronary levels of CRP and MPO did not demonstrate statistical significance (Spearman’s ρ = 0.208, *p* = 0.203). Furthermore, there was no significant positive correlation between coronary CRP with coronary sCD40L (Spearman’s ρ = 0.121, *p* = 0.463) and intracoronary PlGF (Spearman’s ρ = 0.055, *p* = 0.739), as well.

## Discussion

This study demonstrated that the systemic levels of CRP, MPO, sCD40L and PlGF were significantly higher in ACS compared to CSA patients. The role of inflammation in the pathogenesis of myocardial infarction has been targeted in many studies. Numerous investigations have indicated CRP as a risk marker for the prediction of risk for future cardiovascular events [12, 13]. The association of CRP with cardiovascular disease is often related to inflammation. CRP is produced entirely by hepatocytes during the acute inflammatory response. However, several studies have also suggested that atherosclerotic tissue itself can release CRP [14].

Platelets are a source of inflammatory mediators [15]. The activation of platelets following inflammation is a critical component of atherothrombosis process [16]. The lymphocytes actively release sCD40L after platelet stimulation [17, 18], which eventually stimulate vascular endothelial cells inflammation by the secretion of chemokines and cytokines [4].

Myocardial cell damage is not only related to platelet activation and inflammation, but it is also preceded by the recruitment and activation of polymorphonuclear neutrophils. The polymorphonuclear neutrophils release MPO into circulation, resulting in higher MPO level in ACS patients [19, 20]. Myeloperoxidase promotes plaque fibrous cap weakening by activating MMPs [2] and deactivating MMPs inhibitors [3].

C-reactive protein, MPO, PlGF and sCD40L are not solely produced from human coronary plaque. They can be released systemically from other sources such as liver (CRP), circulating neutrophils (MPO), endothelial cells (PlGF) or circulating platelets (sCD40L). Therefore, the elevated markers in ACS patients could be caused by the substantial release from ruptured coronary plaques or produced by the occluded coronary arteries. Besides, there is a possibility that the markers are released systemically from stimulated non-coronary source, reach the coronary plaque site through blood circulation and trigger plaque rupture. In order to investigate the possible site of origin of the markers responsible in the pathogenesis of ACS, both local intracoronary and systemic concentrations of markers were measured, which was the primary aim of this study.

This study showed no significant difference between levels of CRP, MPO and sCD40L in the coronary circulation compared to systemic venous blood, in the ACS patients. Therefore, we speculate that the markers are mainly released from the non-coronary site. The non-coronary source is possibly triggered by a specific precursor prior to the release of the marker, which eventually contribute to the plaque rupture in ACS patients. However, since the time to sample collection after onset of symptom in ACS patients was quite long and up to 48 h, these biomarkers could be carried from coronary local lesion to the systemic circulation, especially if the markers were produced at an early stage of symptom. Hence, future study targeting blood sampling at multiple time intervals after onset of symptom in ACS patients should carried out to answer this hypothesis.

In contrast, the level of PlGF in the coronary circulation was significantly higher than its level in the systemic circulation of ACS patients. Hence, this finding predicted its elevation in ACS patients could be caused by the substantial release from ruptured coronary plaques or produced by the occluded coronary arteries, rather than by non-cardiac origin source. This finding and speculation were further supported by a study conducted by Iwama et al. [21]. This study indicated that vascular tissue, especially the endothelium within the infarct myocardium can substantially produce PlGF during acute myocardial infarction.

C-reactive protein has been detected in human coronary plaques [22, 23]. The generation of CRP and its complement has been reported in atherosclerotic plaque tissue [24], particularly the unstable plaque showed increased expression of CRP protein [25]. Indeed, human coronary artery smooth muscle cells can produce CRP in response to cytokines [14]. Since there was no increased CRP level in the coronary circulation, we hypothesized the increased production of CRP from systemic hepatic cells resulted in the markedly elevation of CRP in ACS patients. The result showed there was a significant decrease in coronary CRP in CSA patients. We may attribute the decrease to local uptake and catabolism of the protein by phagocytes in coronary plaque site. This speculation is supported by immunohistochemistry studies that showed the thrombi at culprit site contained phagocytic white blood cells with the presence of CRP [26].

Numerous studies have demonstrated higher concentration of protein markers in the systemic blood of ACS patients compared to CSA. However, the comparisons between marker concentrations in coronary circulation at the site of lesion with systemic circulation were merely studied. The present study used a particular method to sample blood from coronary arteries. Coronary blood was collected from the coronary circulation at the site of the culprit lesion, where the level of those markers is expected to be the highest if they are released from the ruptured atherosclerotic plaque.

Coronary CRP levels demonstrated a non significant positive correlation with coronary MPO, sCD40L and PlGF levels. The weak correlations suggested the markers reflect distinct signal pathways other than systemic inflammation that eventually contribute to a pro-inflammatory and pro-coagulating environment in the coronary circulation.

Upon released by activated PMNs, MPO transform the plaque into unstable form with large lipid core and thin fibrous cap. Soluble CD40 ligand released after platelet activation trigger an inflammatory response in vascular endothelial cells by the secretion of cytokines and chemokines. In addition to inflammatory properties, sCD40L stabilizes platelet–platelet aggregates and initiates further platelet activation during thrombosis after plaque rupture. PlGF stimulates vascular inflammation by promoting endothelial activation and macrophage recruitment into atherosclerotic lesions.

However, there is limitation in this study. We only managed to obtain coronary blood sample from coronary ostium in CSA patients since the use of export aspiration catheter is not a routine practice in percutaneous transluminal coronary angioplasty (PTCA) procedure for CSA patients.

## Conclusion

In summary, the systemic concentrations of CRP, MPO, sCD40L and PlGF were increased in ACS compared to CSA patients. This study used an advanced sampling technique to sample the blood from the primary site of the culprit lesion in coronary artery and the events happening at the primary site were compared with those in the systemic circulation by comparing the differences in biomarkers at both sites. No significant difference was found between coronary and systemic values of CRP, MPO and sCD40L. The level of PlGF in the coronary circulation was significantly higher than its level in the systemic circulation of ACS patients. Thus, this study hypothesized that the PlGF in the ACS patients could be released from ruptured coronary plaques or produced by the occluded coronary arteries. The coronary levels of the markers established significant correlations with those in systemic circulation. Hence, this study concluded that the concentrations of CRP, MPO, sCD40L and PlGF sampled in the systemic circulation directly reflected those in the coronary circulation.

## Abbreviations

ACS: acute coronary syndrome
CI: confidence interval
CRP: C-reactive protein
CSA: chronic stable angina
ECG: electrocardiogram
EDTA: Ethylenediaminetetraacetic acid
ELISA: enzyme-linked immunosorbent assay
HOCl: hypochlorous acid
IQR: interquartile range
LDL: low-density lipoprotein
MMP: matrix metalloproteinase
MPO: myeloperoxidase
NSTEMI: non-ST-elevation myocardial infarction
PlGF: placental growth factor
sCD40L: soluble CD40 ligand
SD: standard deviation
SPSS: Statistical Product and Service Solutions
STEMI: ST-elevation myocardial infarction
UA: unstable angina
VEGF: vascular endothelial growth factor
